# Relationship Between Leaflets and Root in Normal Aortic Valve Based on Computed Tomography Imaging: Implication for Aortic Valve Repair

**DOI:** 10.3389/fcvm.2021.731440

**Published:** 2021-11-22

**Authors:** Tianyang Yang, Haini Wen, Ismail El-Hamamsy, Qiming Ni, Yanbin Sun, Dan Zhu

**Affiliations:** ^1^Department of Cardiac Surgery, Shanghai Chest Hospital, Shanghai Jiao Tong University, Shanghai, China; ^2^Department of Pharmacy, Shanghai Chest Hospital, Shanghai Jiao Tong University, Shanghai, China; ^3^Department of Cardiovascular Surgery, Mount Sinai Hospital, Icahn School of Medicine at Mount Sinai, New York, NY, United States; ^4^Department of Radiology, Shanghai Chest Hospital, Shanghai Jiao Tong University, Shanghai, China

**Keywords:** aortic root, aortic valve anatomy, leaflet-root mismatch, 3D computed tomography, aortic valve repair

## Abstract

**Objective:** By assessing the normal dimensions and the relationship between the aortic root and leaflets in Chinese population, the objective of this three-dimensional computed tomography (3DCT)-based study was to establish a matching reference for leaflets and aortic root for aortic valve (AV) repair.

**Method:** Electrocardiogram-gated multi-detector CT was performed on 168 Chinese participants with a normal aortic valve. Measurements of the aortic annuli and leaflets were obtained. The correlations between and the ratios of the specific root and leaflet measurements were analyzed. The references for the leaflet and root dimensions were suggested based on geometric height (gH) using a linear regression equation. The utility of the ratios was tested with CT images of 15 patients who underwent aortic valve repair.

**Result:** The mean annulus diameter (AD), sino-tubular junction (STJ) diameter, geometric height (gH), effective height (eH), free margin length (FML), commissural height (ComH), inter-commissural distance (ICD), and coaptation height (CH) were 22.4 ± 1.7 mm, 27.3 ± 2, 0.4 mm, 15.5 ± 1.7 mm, 8.9 ± 1.2 mm, 32.0 ± 3.4 mm, 17.9 ± 1.9 mm, 23.1 ± 2.3 mm, and 3.1 ± 0.6 mm, respectively. The gH/AD, FML/ICD, and eH/ComH ratios were 0.69 ± 0.07, 1.38 ± 0.08, and 0.50 ± 0.07, respectively. The gH correlated with all other leaflet and root measurements (*P* < 0.01), whereas the FML demonstrated a better correlation with ICD compared with gH (R^2^ = 0.75, and R^2^ = 0.37, respectively). The FML/ICD and eH/ComH ratios might be used to assess leaflet-root mismatch and post-repair leaflet billowing.

**Conclusion:** The normal aortic valve measurements based on 3DCT revealed a specific relationship between the root and leaflets; and this will guide the development of an objective method of aortic valve repair.

## Introduction

Aortic valve repair and valve-sparing aortic root procedures have become increasingly popular interventions for tricuspid and bicuspid valve anomalies (Table 1). Unlike the matured surgical technique of mitral valve repair, the standardization and the reproducibility of surgical approaches to aortic valve (AV) repair remain controversial, as they are technically demanding and the surgical principles for re-establishing the natural correlation between the dimensions of all valve components vary with the different institutions (1–5). Given that the dimensions of the leaflets change with aortic root dilatation, correcting the dilated root based on normal value alone, without restoring an appropriate leaflet-root interrelationship, may, otherwise, induce post-repair leaflet billowing (6). Thus, a profound understanding of normal AV anatomy and setting quantitative methods to evaluate leaflet-root mismatch in aortic regurgitation (AR) are of vital importance for AV repair.

**Table 1:** Criteria to perform an aortic valve (AV) repair according to updated guidelines.

**Guideline**	**Indications**	**Recommendation**	**COR**	**LOE**
2021 ESC/EACTS Guidelines for the management of valvular heart disease	Severe aortic regurgitation	Aortic valve repair may be considered in selected patients	IIb	C
	Aortic root or tubular ascending aortic aneurysm	Valve-sparing aortic root replacement is recommended in young patients with aortic root dilation	I	B
2020 ACC/AHA guideline for the management of patients with valvular heart disease	Chronic aortic regurgitation	Valve sparing may be possible in selected patients with favorable valve anatomy who are undergoing surgical replacement of the aortic sinuses and/or ascending aorta	NA	Limited

*COR, class of recommendation; LOE, level of evidence; NA, not applicable*.

Aortic valve is a three-dimensional (3D) anatomical structure and functional apparatus that consists of root annuli (basal ring and STJ) and leaflets. A deep understanding of the 3D configuration of AV is a prerequisite for determining the causes of AR and the surgical approach to AV repair (7–9). In clinical practice, echocardiography is the major imaging modality for evaluating the AV anatomy, and it can be operated at the bedside and is easy to use. However, the spatial resolution of echocardiography is low, and because of software limitation of the company, several crucial parameters cannot be obtained as desired by the operator. Multi-detector 3D computed tomography (3DCT) provides images with high spatial resolution, and customized software is available for post-processing, which enables the visualization and evaluation of specific 3D valve parameters. Therefore, given the above advantages, the 3DCT has been established as the gold standard for pre-operative trans-catheter aortic valve implantation planning (10, 11).

In this study, we aimed to explore the anatomy of the AV as a functional apparatus of the aortic root and leaflets using 3DCT in a large cohort of healthy Chinese. We also aimed to more comprehensively analyze the spatial and functional relationship among key AV components, set the reference for leaflet/root mismatch, and establish critical information about CT applications in patients with AR and aortic root aneurysm related to the indications for AV repair technique.

## Materials and Methods

### Study Population

The CT datasets obtained from adults who visited the Cardiovascular Health Screening Center between 2017 and 2018 were retrospectively analyzed in this study. The inclusive criteria were the following: (1) patients were confirmed to have normal AV function by transthoracic echocardiogram; (2) patients underwent ECG-gated multi-detector CT with contrast enhancement; (3) patients' age > 18 years; (4) the demographic information of the patient were available and complete. Patients with diseases that altered blood flow patterns across the AV or the anatomy of the aortic root (e.g., heart valve and congenital heart diseases, uncontrolled hypertension, Marfan syndrome) were excluded. Two experienced radiologists examined the dataset independently. Patient will only be enrolled in the study when both physicians reached the agreement on a structurally and functionally normal AV with normal size aortic roots.

As a result, the study included 168 adults, 87 men and 81 women. The mean age was 50.2 ± 15.1 years (range, 26–77 years). The enrolled participants were divided into four groups according to age: group 1, 20–35 years (*n* = 39); group 2, 35–50 years (*n* = 43); group 3, 50 to 65 years (*n* = 50); and group 4, 65 to 80 years (*n* = 36). Additionally, from 2017 to 2018, a total of 15 patients with AR underwent AV repair with or without root replacement; both pre-operative and post-operative CT images were analyzed. Baseline characteristics are presented (Table 2). The Institutional Review Board (IRB) or equivalent ethics committee of the Shanghai Chest Hospital approved the study protocol and publication of data. Patient written consent for the publication of the study data was waived by the IRB, as the research involved no more than minimal risk to subjects.

**Table 2:** Pre-operative characteristics of 15 patients.

**Variable**	**Number of patients (mean ± SD)**
Age (years)	55.9 ± 14.7
Male/Female	12/3
Arterial Hypertension	10
Aortic regurgitation mechanism	
Leaflet prolapse	1
Root aneurysm	6
Ascending aorta dilation	8
STJ diameter (mm)	45.9 ± 6.8
Annulus diameter (mm)	26.0 ± 2.9
Aortic regurgitation	
Grade I / II / III / IV	2/1/3/9
Regurgitation jet	
Eccentric / Central	3/12

*STJ, sino-tubular junction; sd, standard deviation*.

### ECG-Gated 3DCT Measurements

All 3DCT data were systematically analyzed using the Osirix software (Version 9.5.1 Geneva, Swiss). Multiplane reconstruction was performed to visualize and measure dimensions of key AV elements outlined by Hagendorff et al. (12). The anatomic structures and the parameters were mainly categorized into two parts from a geometric and functional perspective and measured by two study investigators independently. A video shows how these AV elements were reconstructed and measured using 3DCT images with detailed workflow (Videos 1–8 and Video legends 1–8).

#### Aortic Root Configuration

**Figure 1:**
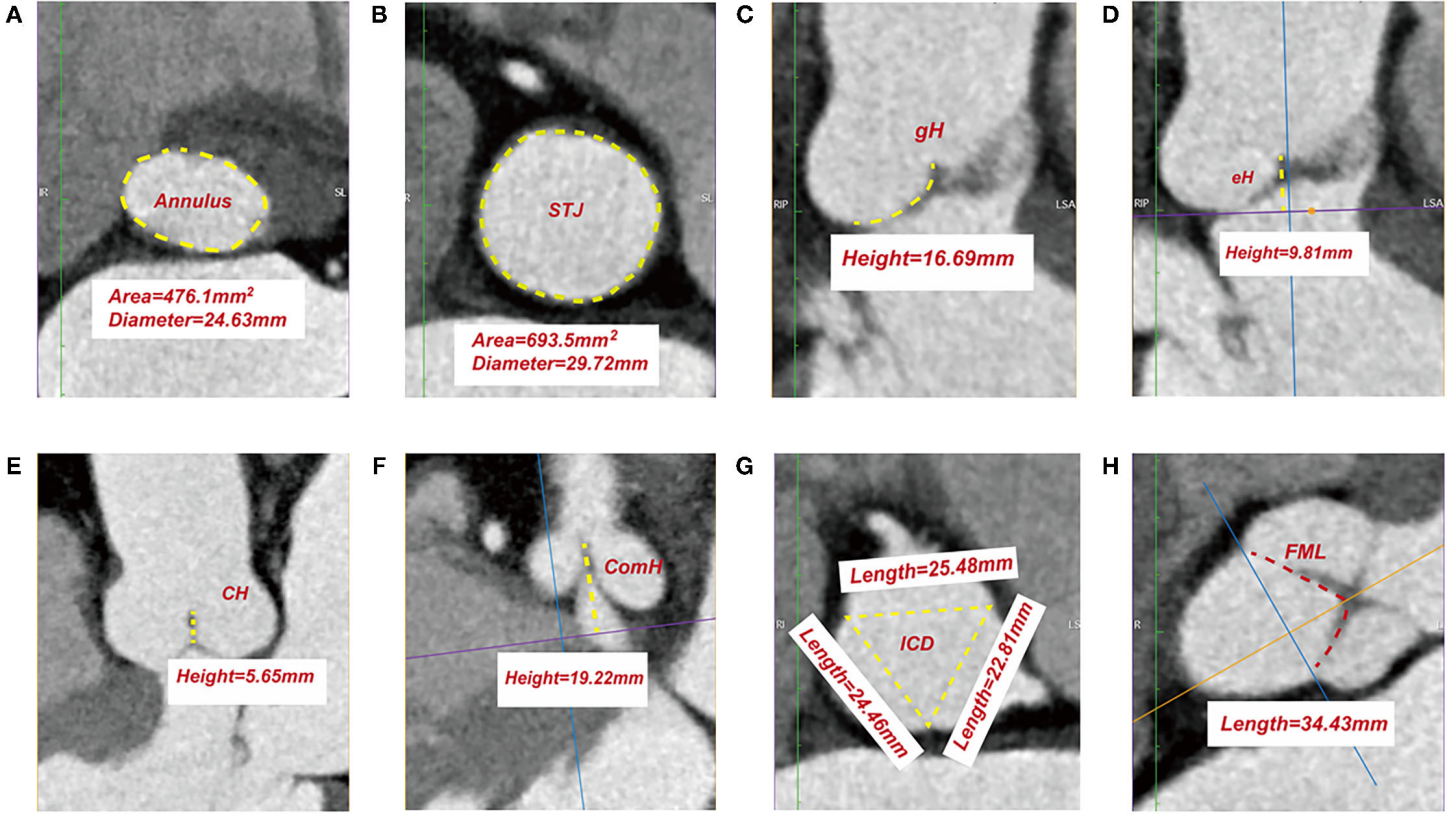
Measurements of leaflet and root parameters in normal aortic valve by three-dimensional reconstruction of ECG-gated computed tomography. Surface area and diameter of annulus **(A)**. Surface area and diameter of sino-tubular junction **(B)**. Measurement of geometric height **(C)**. Measurement of effective height **(D)**. Measurement of coaptation height **(E)**. Measurement of commissure height **(F)**. Measurement of inter-commissural distance **(G)**. Measurement of free margin length **(H)**. STJ, sino-tubular junction; gH, geometric height; eH, effective height; CH, coaptation height; ComH, commissure height; ICD, inter-commissural distance; FML, free margin length.

Two-dimensional measurements of the aortic root were made at end-diastole (80% RR interval) at these levels of the functional annuli: (1) annulus or basal ring (determined by the transverse plane crossing the nadirs of the leaflets, Video 1) and (2) sino-tubular junction (STJ, determined by the narrowest plane connecting the sinus and ascending aorta, Video 8). The following parameters were obtained: annulus and STJ surface area, annulus diameter (AD) and STJ diameter (derived using the surface area under the assumption that the annulus and STJ were circular), STJ area to annulus area ratio (STJ/Annulus area), and STJ diameter to annulus diameter ratio (STJ/AD) (Figures 1A,B).

#### Leaflet Geometry

Leaflet geometries were only assessed at end-diastole (80% RR interval), during which the image artifact resulting from the vibration of the leaflets is least, and the closure of the AV is best evaluated with leaflets fully pressurized by the diastolic pressure. The leaflet parameters investigated are as follows (most definitions and nomenclatures are by Hagendorff et al.) (12): geometric height (gH), referring to the distance between the curved length of the respective cusp during diastole from the aortic insertion in the nadir of the sinus to the central part of the free margin (Video 2); effective height (eH), referring to the difference in height between the annular plane and the free margin of each cusp during diastole (Video 3); coaptation height (CH, Video 4); free margin length (FML, Video 7); commissure height (ComH), referring to the distance between the commissural apex and the annular plane (Video 5); inter-commissural distance (ICD), referring to the distance between the two adjacent commissural apices (Video 6) (Figures 1C–H).

#### Leaflet-Root Ratios

The ratios of the average gH to the calculated AD (gH/AD), average FML (gH/FML), average eH (eH/gH), and calculated STJ diameter (gH/STJ); the ratio of the average FML to the average ICD (FML/ICD); and the ratio of the average eH to the average ComH (eH/ComH) of the leaflets were determined in our study to demonstrate the relationship between the leaflet and root geometries.

### Pre- and Post-repair CT Images of 15 Patients With AR

All 15 patients enrolled for analysis underwent ECG-gated 3DCT within 1 month prior to the AV repair; CT scan was repeated to obtain post-repair images between post-operative days 7 and 10. The aforementioned measurements and leaflet-root ratios were obtained to evaluate pre-operative AR mechanism and to assess the post-repair outcome.

### Statistical Analyses

Continuous variables were assessed using the Kolmogorov-Smirnov test for normal distribution and presented as the mean ± standard deviation. Independent-sample *t*-test or one-way ANOVA was used to compare the means of the groups. Linear regression was performed to calculate the Pearson correlation coefficient (R), and a *p* < 0.05 was considered statistically significant. Linear regression equations were constructed using gH as the variable and the other parameters as dependent variables. The R statistical software version 3.6.1 was used to perform all the statistical analyses. For all the parameters derived from the 3DCT measurements, the two independent observers achieved inter-observer variability of 0.909–0.987 (intra-class correlation coefficients) and an intra-observer variability of 0.920–0.989. All measurements were <10% of coefficient of variation (range, 4.2–9.1%).

## Results

### Normal Aortic Valve

The measurements, with their ranges and means, for the normal AV are reported based on root configuration and leaflet geometries, and the data are stratified by age and gender.

For the normal AV, the average annulus area was 396.1 ± 60.3 mm^2^ (range, 278.3–516.0 mm^2^), the area-derived AD was 22.4 ± 1.7 mm (range, 18.8–25.6 mm), the average STJ area was 590.5± 101.7 mm^2^ (range, 349.8–771.9 mm^2^), the area-derived STJ diameter was 27.3 ± 2.4 mm (range, 21.1–31.4 mm), the STJ/annulus area ratio was 1.5 ± 0.2 (range, 1.0–2.1), the STJ/AD ratio was 1.2 ± 0.1 (range, 1–1.4), the average gH was 15.5 ± 1.7 mm (range, 11.0–21.6 mm) with the highest mean gH (16.2 ± 1.6 mm) in the non-coronary (NC) leaflet and the shortest in the right coronary (RC) leaflet (14.9 ± 1.7 mm), the mean eH was 8.9 ± 1.2 mm (range, 6.29–12.0 mm), and the three leaflets showed no significant difference (*p* = 0.867). The mean FML and ICD, all being longest in the RC and shortest in the left coronary (LC) leaflet, were 32.0 ± 3.4 mm (range, 21.3–40.7 mm) and 23.1 ± 2.3 mm (range, 17.4–28.2 mm), respectively. The mean ComH, being longest between the NC and RC leaflets, was 17.9 ± 1.7 mm (range, 13.0–23.7 mm). The mean CH, being longest for NC leaflets, was 3.1 ± 0.6 mm (range, 1.33–5.26 mm) (Supplementary Tables S1, S2).

Specific ratios were established to elucidate the spatial relationship between the leaflets and root geometries; the average gH/AD, gH/FML, eH/gH, FML/ICD, and eH/ComH were 0.69 ± 0.07, 0.49 ± 0.06, 0.58 ± 0.07, 0.57 ± 0.05, 1.38 ± 0.08, and 0.50 ± 0.07, respectively. The age groups showed no statistically significant difference (Supplementary Table S3). The absolute values of the root and leaflet parameters were significantly greater in men than in women at all levels (*p* < 0.001), but the same leaflet-root ratios were maintained. Although no significant differences were found among the age groups for most parameters, the trends of greater STJ/annulus ratios were observed along with an increase in age (*p* < 0.01) (Supplementary Table S4).

**Figure 2:**
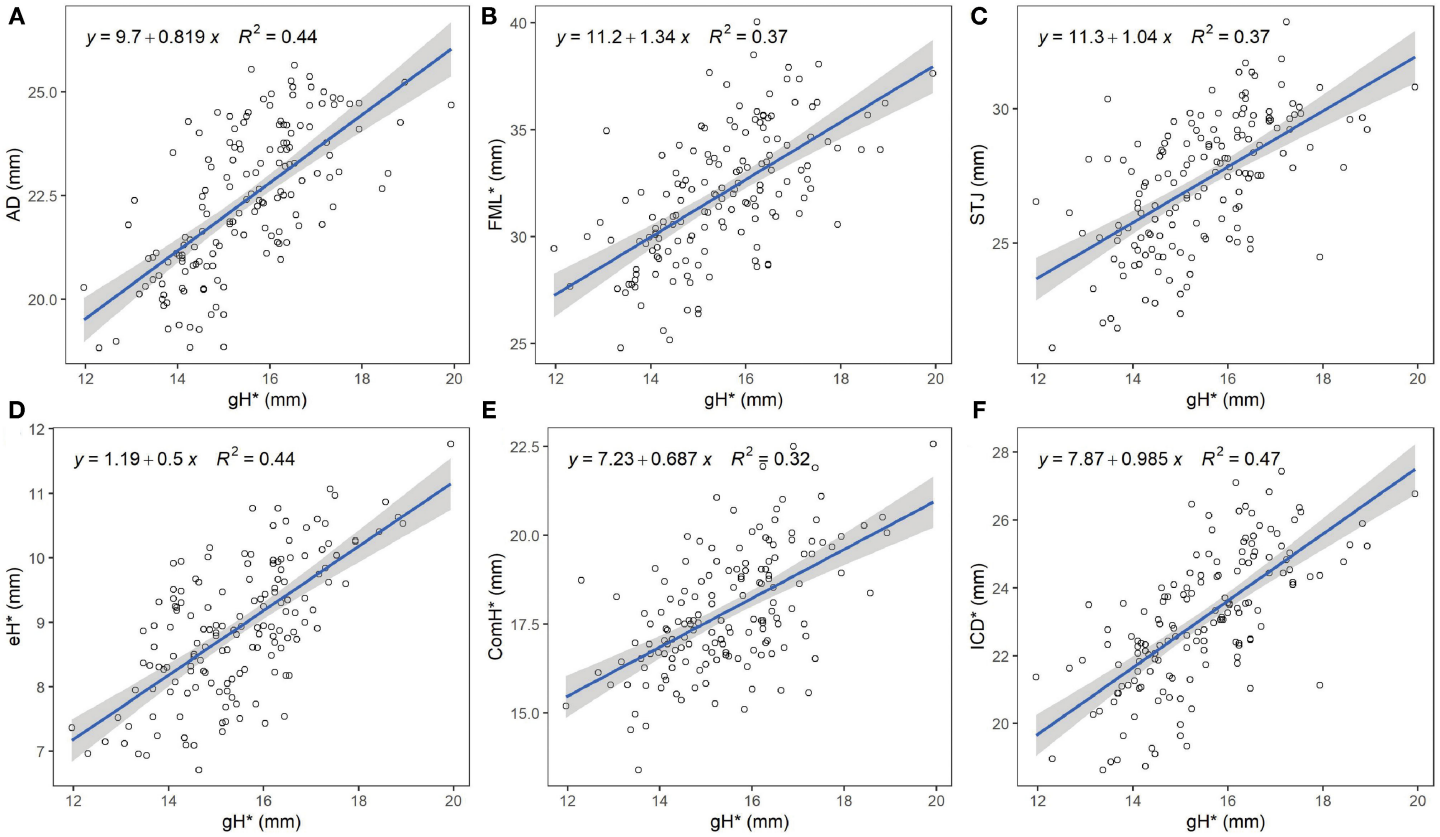
Relationship between geometric height and aortic valve measures. The blue line represents the best fit linear regression model of the data points. The shaded gray area represents the 95% confidence level interval for predictions from each linear model. **(A)** Linear regression between gH and AD. **(B)** Linear regression between gH and FML. **(C)** Linear regression between gH and STJ area-derived diameter. **(D)** Linear regression between gH and eH. **(E)** Linear regression between gH and ComH **(F)** Linear regression between gH and ICD. gH, geometric height; AD, annular diameter; FML, free margin length; STJ, sino-tubular junction; eH, effective height; ComH, commissural height; ICD, inter-commissural distance. *, average measures of three leaflets.

**Figure 3:**
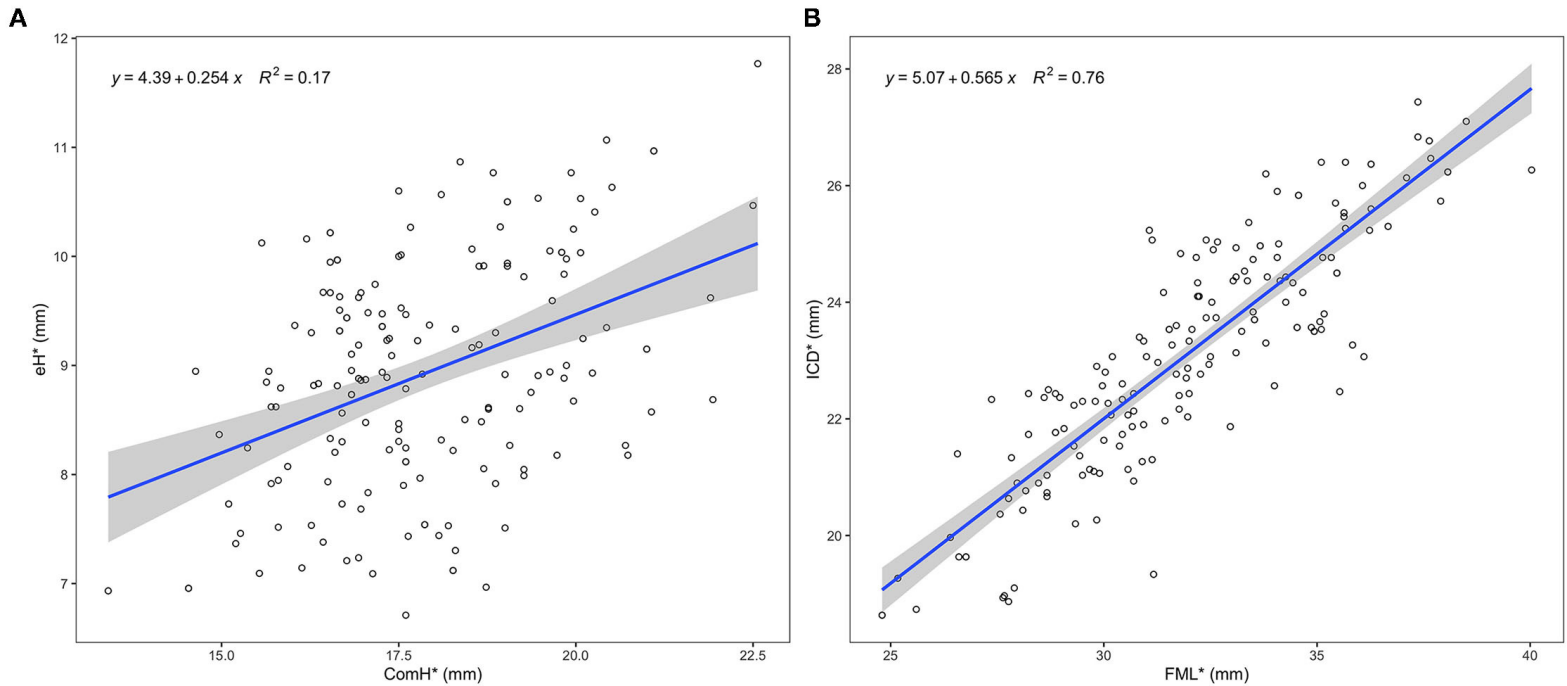
Relationship between specific leaflet and root measures. The blue line represents the best fit linear regression model of the data points. The shaded gray area represents the 95% confidence level interval for predictions from each linear model. **(A)** Linear regression between ComH and eH. **(B)** Linear regression between FML and ICD. eH, effective height; ComH, commissural height; FML, free margin length; ICD, inter-commissural distance; *, average measures of three leaflets.

All the measured leaflet and root dimensions showed positive correlations with gH (*p* < 0.0001), whereas FML demonstrated a better correlation with ICD compared with gH (R^2^ = 0.75, R^2^ = 0.37). eH was also positively correlated with ComH but not as well as with gH (R^2^ = 0.16, R^2^ = 0.44) (Figures 2, 3). Based on the gH ranging from 12 to 25 mm, we calculated the related leaflet and root parameters using linear regression equations (Supplementary Table S5).

### Cohort of 15 AR Patients With Pre- and Post-repair CT Images

**Figure 4:**
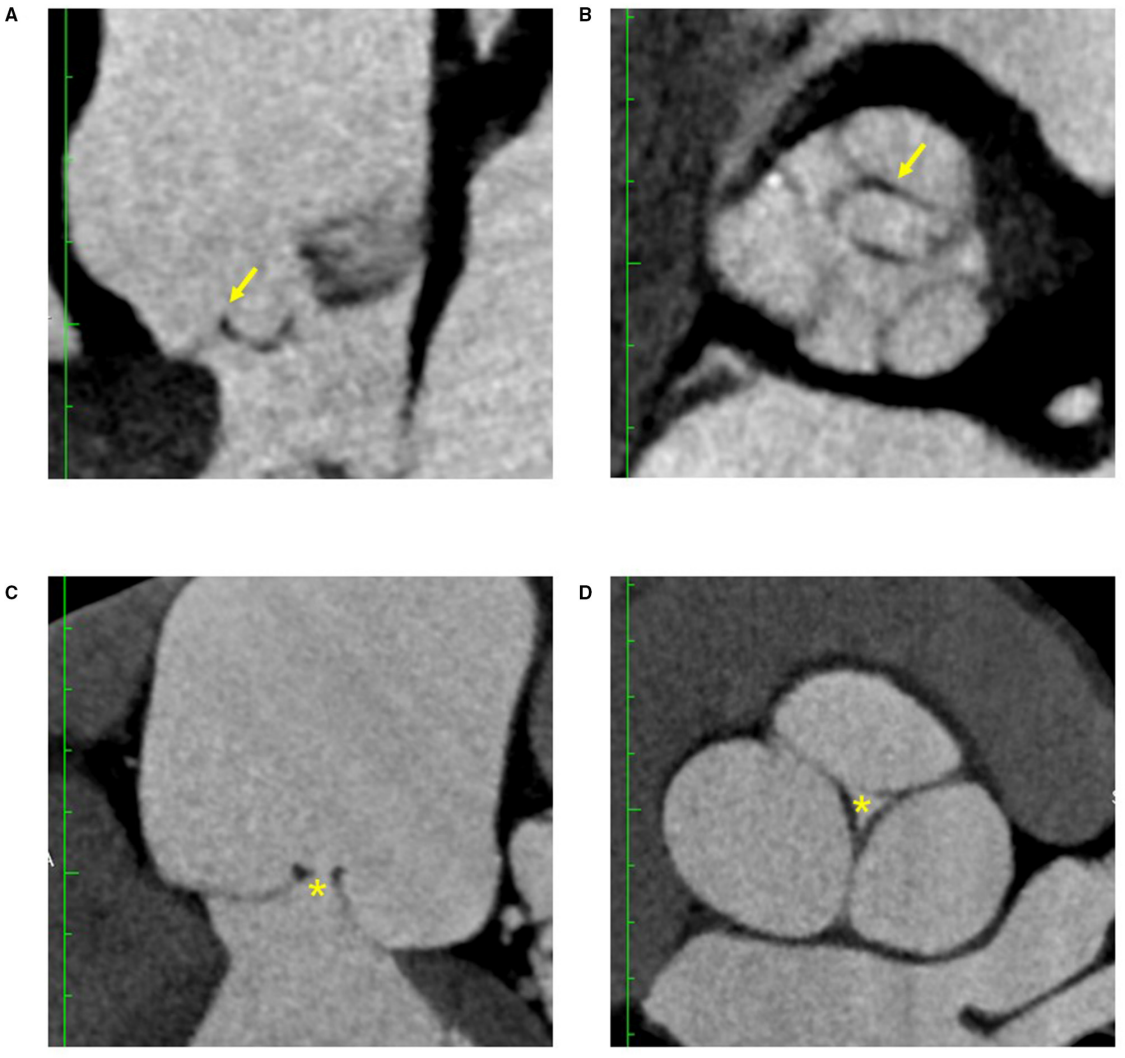
CT features of AR with eccentric jet and central jet. Bent leaflet belly indicated by arrows were both identified in long axis **(A)** and short axis **(B)** of reconstructed CT images in AR with eccentric jet. Loss of coaptation indicated by asterisks were both identified in long axis **(C)** and short axis **(D)** of the reconstructed CT images in AR with central jet. AR, aortic regurgitation.

**Figure 5:**
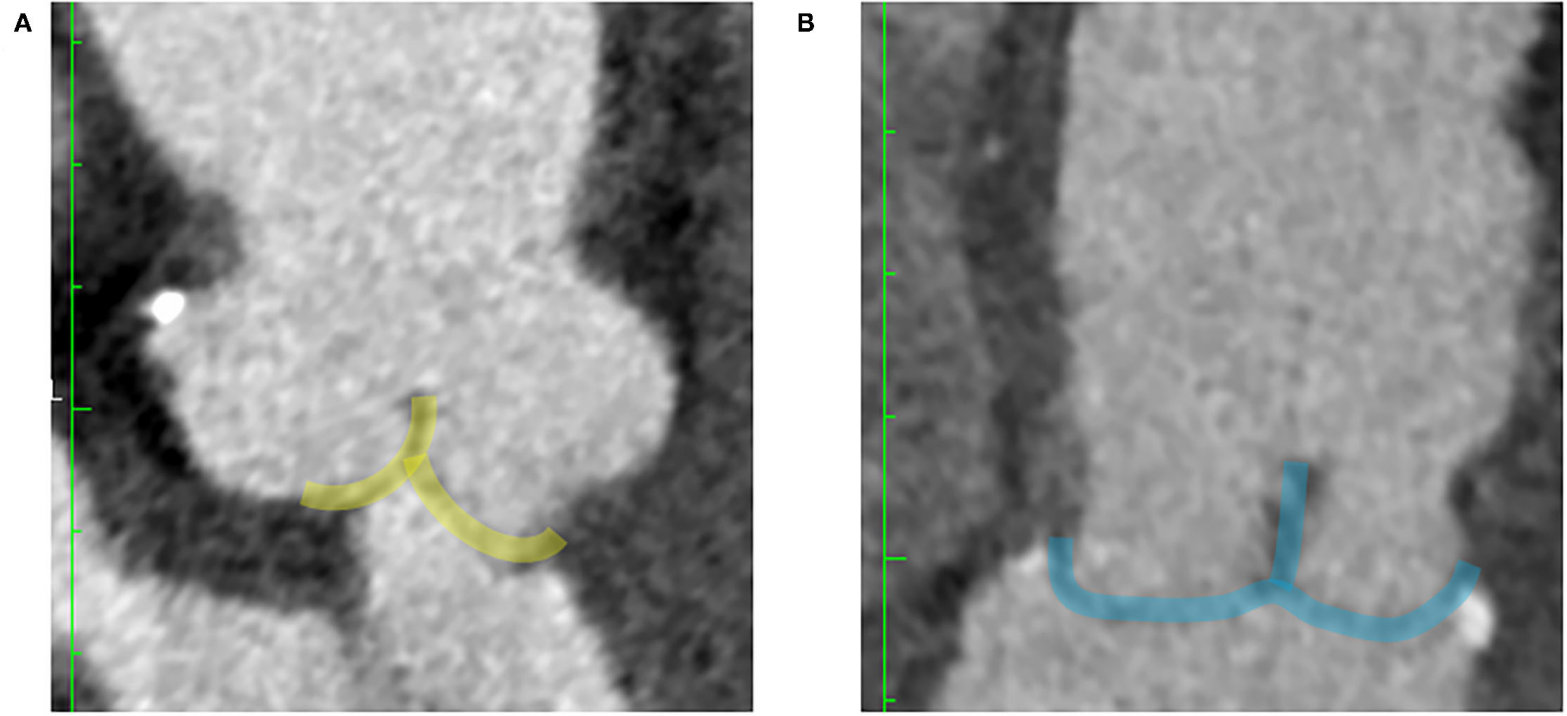
CT features of post-repair valve coaptations. The shaded green area delineated the post-repair coaptation without leaflet billowing in a reversed Y form **(A)**. The shaded blue area delineated the post-repair coaptation with leaflet billowing in a reversed T form **(B)**.

The three patients with leaflet prolapse causing eccentric AR jet were identified by bent leaflet belly in the pre-repair CT images, which was not presented in the other 12 patients with central jet (Figure 4). After AV repair, leaflet billowing without prolapse defined by altered coaptation configuration from normal reversed “Y” to reversed “T” shape in the post repair CT images was found in five patients (Figure 5). The post-operative echocardiogram demonstrated no more than mild AR in all 15 patients. The detailed valve parameters and leaflet-root ratios based on CT images were measured and calculated in pre- and post-repair groups (Table 3).

**Table 3:** Statistical comparison and detailed pre- and post-operative leaflet-root parameters of 15 aortic valve repair cases.

**Case**	**STJ (mm)**		**AD (mm)**		**FML/ICD**		**gH/AD**		**eH mm**		**eH/ComH**	
**No.**	**(pre vs. post)**		**(pre vs. post)**		**(pre vs. post)**		**(pre vs. post)**		**(pre vs. post)**		**(pre vs. post)**	
1*	50.6	29.0	26.8	25.9	1.28	1.61	0.72	0.73	10.3	8.0	0.46	0.37
2	36.2	25.5	21.8	21.1	1.14	1.28	0.77	0.78	10.8	8.8	0.69	0.55
3^#^	47.6	34.9	27.6	24.9	1.38	1.39	0.77	0.86	11.2	13.7	0.46	0.56
4*	43.1	24.2	24.5	19.6	1.30	1.67	0.65	0.81	10.7	7.7	0.52	0.43
5*	53.5	30.3	23.9	20.3	1.11	1.60	0.87	1.01	15.4	9.4	0.67	0.43
6	53.3	31.4	27.6	23.4	1.15	1.43	0.70	0.84	13.9	10.5	0.63	0.48
7*	52.7	31.5	31.6	26.4	1.28	1.45	0.75	0.88	14.4	10.2	0.54	0.39
8^#^	43.3	31.2	29.3	22.7	1.38	1.40	0.58	0.75	8.4	9.2	0.43	0.46
9^#^	42.6	28.9	29.2	24.0	1.45	1.41	0.69	0.82	6.9	10	0.32	0.46
10	44.2	30.2	21.6	21.7	1.20	1.45	0.92	0.90	15.4	10.2	0.67	0.46
11	40.4	29.6	27.0	24.3	1.21	1.32	0.69	0.77	11.5	10.2	0.66	0.55
12	51.5	31.4	28.2	25.9	1.32	1.37	0.76	0.82	14.7	12.2	0.59	0.51
13*	57.1	28.9	24.7	24.7	1.35	1.58	0.76	0.73	11.0	6.3	0.39	0.26
14	33.5	27.3	23.3	22.9	1.13	1.35	0.80	0.76	11.7	9.1	0.70	0.59
15	39.4	29.6	22.8	21.3	1.25	1.31	0.65	0.69	10.5	9.8	0.58	0.55
mean	45.9	29.6	26.0	23.3	1.26	1.44	0.74	0.81	11.7	9.7	0.55	0.47
sd	7.0	2.6	3.0	2.1	0.10	0.12	0.09	0.08	2.5	1.8	0.12	0.09
*p*	<0.001		<0.001		<0.001		0.002		0.008		0.008	

*STJ, sino-tubular junction (area-derived diameter); AD, annulus diameter; FML, free margin length; ICD, inter-commissural distance; Gh, geometric height; eH, effective height; ComH, commissure height; Pre, pre-repair, post, post-repair; sd, standard deviation*.

# *, cases with pre-repair eccentric jet*;

* *, cases with post-repair leaflet billowing*.

The valve parameters and leaflet-root ratios were markedly different between pre- and post-repair group. The 15 patients were divided into two groups based on classification of AR jet (eccentric vs. central) pre-operatively, and they were categorized into two groups according to post-repair leaflet billowing post-operatively.

Pre-operatively, the patients with central jet showed significantly smaller FML/ICD ratio than patients with eccentric jet (1.23 ± 0.08 vs. 1.40 ± 0.04, *p* = 0.001). Both eH and eH/ComH were smaller in the eccentric jet group, but significance was only observed for eH/ComH (*p* = 0.021). Meanwhile, eccentric jet group showed significantly larger AD than central jet group (28.7 ± 1 vs. 25.3 ± 3 mm, *p* = 0.007) (Table 4).

**Table 4:** Pre-repair leaflet-root parameters stratified by AR jet.

**Pre-repair parameters**	**Eccentric jet**	**Central jet**	***p***
	***N*** **= 3**	***N*** **= 12**	
FML/ICD	1.40 ± 0.04	1.23 ± 0.08	0.001
gH/AD	0.68 ± 0.10	0.75 ± 0.08	0.314
eH/ComH	0.40 ± 0.07	0.59 ± 0.10	0.021
eH (mm)	8.8 ± 2.2	12.5 ± 2.0	0.078
STJ (mm)	44.5 ± 2.7	46.3 ± 7.8	0.527
AD (mm)	28.7 ± 1.0	25.3 ± 3.0	0.007

*AR, aortic regurgitation; STJ, sino-tubular junction (area-derived diameter); AD, annulus diameter; FML, free margin length; ICD, inter-commissural distance; gH, geometric height; eH, effective height; ComH, commissure height*.

Post-operative FML/ICD ratios were much larger in the patients showing post-repair leaflet billowing than in those without billowing (1.58 ± 0.08 vs. 1.37 ± 0.06, *p* = 0.002). Both eH and eH/ComH were smaller in the billowing group (Table 5).

**Table 5:** Post-repair leaflet-root parameters stratified by leaflet billowing.

**Post-repair parameters**	**Billowing**	**Without billowing**	***p***
	***N*** **= 5**	***N*** **= 10**	
FML/ICD	1.58 ± 0.08	1.37 ± 0.06	0.002
gH/AD	0.83 ± 0.12	0.80 ± 0.06	0.581
eH/ComH	0.38 ± 0.07	0.52 ± 0.05	0.007
eH mm	8.3 ± 1.5	10.4 ± 1.5	0.039
STJ mm	28.8 ± 2.8	30.0 ± 2.5	0.435
AD mm	23.4 ± 3.2	23.2 ± 1.6	0.920

*STJ, sino-tubular junction (area-derived diameter); AD, annulus diameter; FML, free margin length; ICD, inter-commissural distance; gH, geometric height; eH, effective height; ComH, commissure height*.

## Discussion

Our study aimed to comprehensively explore the anatomy of normal tricuspid AV with 3DCT, with a focus on the relationship between leaflet geometry and functional root annuli dimension *in vivo*. CT data were also collected on a cohort of 15 patients who underwent AV repair to assess potential implication of specific leaflet-root relationship. Several studies have already reported the dimensions of normal AV based on *ex vivo* evaluations of the root specimens (13–15), without physiologic blood pressurization; thus, the leaflet-root relationship has not been thoroughly established and requires further clarification. The development of cardiac CT and dedicated software for post-processing allows for a comprehensive 3D assessment of AV *in vivo* with improved spatiotemporal resolution (16). Moreover, these measurements can be acquired robustly with proposed workflow, and both built-in CT workstation and commercialized software can be utilized with minimal measurement variations.

The configurations of the leaflet and root of the normal aortic valve are asymmetric. Consistent with the findings of previous studies (17, 18), leaflet asymmetry was also confirmed in our study. We took a further step with 3DCT analysis; we took more measurements of the leaflets in the normal AV and found that they were asymmetrical at multiple geometric levels. The shortest gH and the longest FML of the RC leaflet make it slender than the NC and LC leaflets, which could be the cause of the more frequent prolapse of the RC leaflet in AR. On the other hand, the longest ICD of the RC leaflet may be responsible for ensuring an eH that is equivalent to those of the NC and LC leaflets, as revealed by our study. This suggested that a specific leaflet-root ratio, which was evaluated but not fully validated by small a sample study by De Kerchove et al. (15), may exist at the STJ level to maintain valve competency. Similarly, the ComHs were also uneven, being highest between the NC and RC leaflets and lowest between the NC and LC leaflets, and this resulted in the tilting of the aortic root with non-parallel STJ and basal ring (19). Based on the above *in vivo* 3DCT findings, the current AV repair strategy does not seem to consider the natural AV asymmetry by positioning the ComHs symmetrically, both circumferentially and longitudinally, within the Dacron graft during valve-sparing root procedures.

Leaflet remodeling and adaption in aortic root dilation have been confirmed by multiple studies (16, 18, 20), and the gH and FML, as important leaflet sizing parameters, were found involved in this adaptive process. The size of the native leaflet indicated by gH is the major constraining factor for a successful AV repair without patch augmentation, which makes gH an important sizing parameter (21). Being another key element of the leaflet dimension, FML is closely associated with leaflet adaption and prolapse in the cases of root dilation with or without AR (15, 22, 23). Leaflet adaptions, along with root dilation, which commonly presents as the elongation of FML and an increase in gH (16, 20, 24), have not been fully addressed in AV repair due to frequent leaflet billowing after root replacement or annuloplasty despite CH restoration (6). Current AV repair strategies do not sufficiently consider the leaflet-root relationship, which may lead to over- or under-correction by targeting eH at a fixed value (eH > 9 mm) (25, 26). Over reduction of STJ in root dilation with an elongated FML may also require excessive leaflet plication resulting in a “large leaflet in a small root,” and long-term hemodynamics under these circumstances is not ideal. Furthermore, using gHs of <16 mm as a relative contraindication for AV repair may need reconsideration, given the small habitus in Asian group (21, 27). Therefore, we established the leaflet and root measurements based on gH using a linear regression equation to guide the AV repair and improve the practicality of the current study for a wider range of gH values (12–25 mm) (Table 5). Additionally, the better correlation of FML with ICD than with gH (Figure 5) implies that an independent approach may be necessary for free-margin plication and STJ reconstruction for better post-repair valve configurations. We hope that the development of dedicated sizers will improve the usefulness of the above measurement.

**Figure 6:**
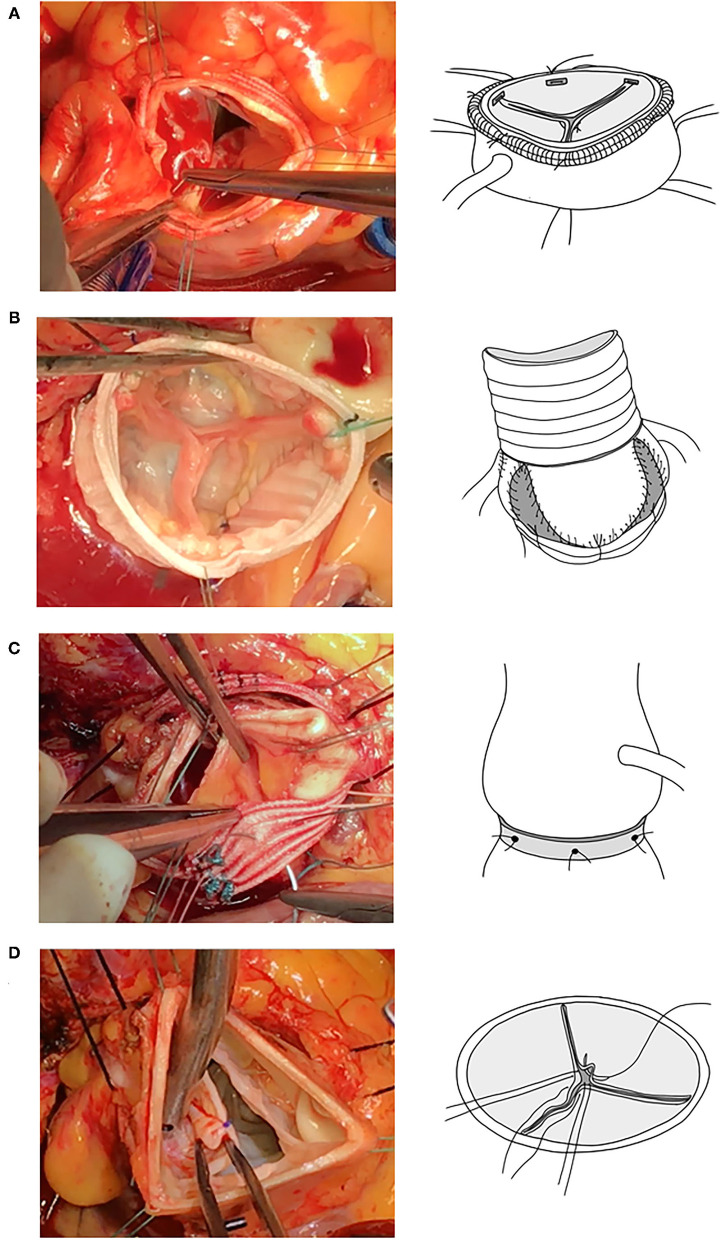
Important current AV repair techniques. From top downward (left column, surgical views; right column, illustrative figures), STJ remodeling **(A)**, valve sparing root replacement **(B)**, annuloplasty **(C)**, and FML central plication **(D)**. AV, aortic valve; STJ, sino-tubular junction; FML, free margin length.

The classification of functional aortic leaflet-root abnormality was established by El Khoury to guide the assessment of AR mechanism and AV repair for the tricuspid AV (Table 6) (28). Based on our normal AV findings, we attempted to define these abnormalities as leaflet-root mismatches and suggest further divisions, based on the 3DCT measurements, into leaflet-STJ mismatch defined as FML/ICD ratio of 1.38 and leaflet-basal ring mismatch defined as gH/AD ratio of 0.69 derived from the mean value of normal population. The goal of AV repair is to achieve sufficient CH and adequate eH proportional to ComH defined as eH/ComH ratio of 0.5 (29, 30), which can be translated into restoring a normal gH/AD ratio by basal ring annuloplasty to correct annular ectasia and a normal FML/ICD ratio by combining FML plication and STJ reconstruction to correct leaflet prolapse and STJ dilation (Figure 6). Based on these normal ratios, we retrospectively analyzed the pre- and post-operative 3DCT images of 15 patients who underwent AV repair at our center without applying the matching methodology as suggested by the current study, and the results showed that the deviation from the normal reference values of the key leaflet-root ratios can be used for pre-operative AR assessment and post-operative review of the technique-related leaflet billowing.

**Table 6:** AV repair techniques based on the classification of AR with description of disease mechanisms.

**AR class**	**Type I**			**Type II**
	**Normal leaflet motion with annuli dilatation**			**Increased leaflet motion**
	**Ia**	**Ib**	**Ic**	
Mechanism	STJ dilation	Root dilation ± STJ dilation	Annulus dilation	Leaflet prolapse
Repair technique	STJ remodeling or ascending aortic graft	Valve sparing root replacement	Annuloplasty	FML central plication

*AR, aortic regurgitation; STJ, sino-tubular junction; FML, free margin length*.

When normal leaflet-root ratios were referred for pre-operative AR assessment, patients of our cohort with central AR featured a reduced FML/ICD ratio (1.28) and increased the eH/ComH ratio (0.59), probably indicating FML tethering and relative diastolic restrictive motion secondary to insufficient leaflet remodeling in relation to root dilatation. In contrast, the FML/ICD ratio (1.40) was increased and the eH/ComH ratio (0.4) was decreased in the case of eccentric AR resulted from leaflet prolapse, rather expressing excessive leaflet motion.

In terms of assessment of post-repair leaflet billowing, it was featured by a significantly increased FML/ICD ratio (1.58) and a reduced eH/ComH ratio (0.38). This phenomenon could be due to the over-reduction of STJ which led to leaflet redundancy. Although central plication of free margin was performed routinely to restore normal eH, the post-repair eH was still insufficient for the dilated root to reach the eH/ComH ratio of 0.5. In contrast, it can be seen from those cases without post-repair leaflet billowing that no leaflet-root mismatch was defined by altered ratios; both FML/ICD (1.37) and eH/ComH (0.52) ratios were comparable to normal population.

## Study Limitation

We did not investigate the impact of age and body surface area on AV configuration, as have been discussed extensively in the literature (7, 14, 31–33), although they have important effects on AV repair. Because of the small habitus of our cohort compared with that of the western population, the gH measurements did not cover the 22–25 mm range, which limits the application of the gH-based ratio. The extreme gH measurements (<12 mm) also need to be validated. The calculated leaflet and root parameter reference values for AV repair also require further validation. The retrospective approach to analysis of the application of leaflet-root ratio and the low number of patients in a small cohort of 15 patients were the major limitations. The analyzed dataset of normal AV valves was retrospectively enrolled by two independent physicians, based on the CT images and medical records of the patients. While the data might be biased, they can still reflect leaflet-root relationships as potential reference for AV repair, especially when normal anatomy is absent. Extensive application of CT to perioperative root assessment could also be limited due to ionizing radiation. Comparative studies of the pre- and post-AV repair 3DCT images of AR patients with large samples are needed for further exploration. All measurements were performed based on 3DCT without validation by direct comparison *via* surgical inspection.

## Conclusion

We described the normal dimensions of the aortic root configuration and leaflet geometry in the tricuspid AV based on 3DCT findings. Moreover, the findings from this study on the relationship between the leaflet and root with further investigations into the key geometrical features of valve closure may help improve the techniques for restoring normal functional anatomic relationships of pathologically altered aortic roots. A dedicated sizing parameter for assessing key functional elements with proper indexation, such as gH-indexed AD, FML-indexed STJ, and ComH-indexed eH, may be developed to standardize leaflet repair for root pathology.

## Data Availability Statement

The raw data supporting the conclusions of this article will be made available by the authors, without undue reservation.

## Ethics Statement

The studies involving human participants were reviewed and approved by the Institutional Review Board (IRB) or Equivalent Ethics Committee of the Shanghai Chest Hospital. Written informed consent for participation was not required for this study in accordance with the national legislation and the institutional requirements.

## Author Contributions

TY, DZ, and IE-H had the idea for the study design and critically corrected the manuscript. QN and YS were involved in data collection and data verification. TY and HW drafted the manuscript. All authors read and approved the final manuscript.

## Funding

The study was supported by the grant from Shanghai Municipal Commission of Health and Family Planning (20184Y0076 to TY) and the grant from Shanghai Hospital Development Center (SHDC12020124 to DZ).

## Conflict of Interest

The authors declare that the research was conducted in the absence of any commercial or financial relationships that could be construed as a potential conflict of interest.

## Publisher's Note

All claims expressed in this article are solely those of the authors and do not necessarily represent those of their affiliated organizations, or those of the publisher, the editors and the reviewers. Any product that may be evaluated in this article, or claim that may be made by its manufacturer, is not guaranteed or endorsed by the publisher.

## Abbreviations

AV: aortic valve
AD: annulus diameter
AR: aortic regurgitation
CH: coaptation height
ComH: commissure height
3DCT: three-dimensional computed tomography
eH: effective height
FML: free margin length
gH: geometric height
ICD: inter-commissural distance
LC: left coronary leaflet
NC: non-coronary leaflet
RC: right coronary leaflet
STJ: Sino-tubular junction.
