# Supplementary material for: Relationship Between Leaflets and Root in Normal Aortic Valve Based on Computed Tomography Imaging: Implication for Aortic Valve Repair
Source: Front Cardiovasc Med. 2021 Nov 22;8:731440. doi: 10.3389/fcvm.2021.731440 (PMC8645849; doi:10.3389/fcvm.2021.731440)
Supplement: Supplementary file 1 [file Data_Sheet_1.docx]

Table S1. Statistical comparison of root measures and ratios between age groups

| Root Parameters | Age1  (n=39) | 2  (n=43) | 3  (n=50) | 4  (n=36) | Mean | *p* |
| --- | --- | --- | --- | --- | --- | --- |
| Annulus surface area  mm^2^ | 407.8 ± 62.5 | 405.5 ± 65.1 | 384.5 ± 57.0 | 388.2 ± 54.3 | 396.1±60.3 | 0.175 |
| Annulus diameter (area derived) mm | 22.7 ± 1.8 | 22.7 ± 1.8 | 22.1 ± 1.6 | 22.2±  1.6 | 22.4 ±  1.7 | 0.196 |
| STJ surface area mm^2^ | 568.6 ± 109.1 | 584.3 ±  99.2 | 588.9 ±  104.0 | 623.9 ± 88.1 | 590.5 ± 101.7 | 0.117 |
| STJ diameter (area derived) mm | 26.8±  2.6 | 27.2 ±  2.4 | 27.3 ± 2.4 | 28.1 ± 2.0 | 27.3 ± 2.4 | 0.105 |
| STJ/Annulus area | 1.4 ± 0.2 | 1.5 ± 0.2 | 1.5 ±  0.2 | 1.6 ±  0.2 | 1.5±  0.2 | <0.001 |
| STJ/AD | 1.18± 0.09 | 1.20 ± 0.09 | 1.24 ± 0.09 | 1.27 ± 0.07 | 1.22 ± 0.09 | <0.001 |

STJ: sino-tubular junction; AD: annulus diameter

Table S2. Inter and intra age groups statistical comparison of leaflet measures

| Leaflet Parameters | Age 1  (n=39) | 2  (n=43) | 3  (n=50) | 4  (n=36) | Mean | *p* |
| --- | --- | --- | --- | --- | --- | --- |
| gH mm |  |  |  |  |  |  |
| NC | 16.3±1.4 | 16.3±1.3 | 15.9±1.6 | 16.5±1.9 | 16.2±1.6 | 0.286 |
| RC | 15.3±1.8 | 15.0±1.6 | 14.4±1.6 | 14.7±1.7 | 14.9±1.7 | 0.058 |
| LC | 15.6±1.6 | 15.4±1.4 | 15.2±1.6 | 15.6±1.9 | 15.4±1.6 | 0.540 |
| Mean | 15.8±1.7 | 15.6±1.5 | 15.2±1.7 | 15.6±1.9 | 15.5±1.7 | 0.198 |
| *p* | 0.020 | <0.001 | <0.001 | 0.001 | <0.001 |  |
| eH mm |  |  |  |  |  |  |
| NC | 9.0±1.1 | 9.0±1.3 | 8.9±1.1 | 8.9±1.2 | 8.9±1.2 | 0.964 |
| RC | 8.9 ±1.2 | 9.0±1.2 | 8.8±1.0 | 8.9 ±1.3 | 8.9±1.2 | 0.796 |
| LC | 8.7±1.0 | 9.0±1.1 | 8.9 ±1.2 | 9.2±1.2 | 9.0±1.2 | 0.448 |
| Mean | 8.9±1.1 | 9.0±1.2 | 8.8±1.1 | 9.0±1.2 | 8.9±1.2 | 0.879 |
| *p* | 0.570 | 0.968 | 0.765 | 0.648 | 0.867 |  |
| ComH mm |  |  |  |  |  |  |
| NR | 18.8±2.1 | 18.1±2.1 | 18.3±2.0 | 18.4±2.0 | 18.4±2.0 | 0.510 |
| RL | 18.5±1.9 | 17.7±1.9 | 17.5±1.7 | 17.9±1.8 | 17.9±1.9 | 0.114 |
| LN | 17.8±1.8 | 17.0±1.6 | 17.2±1.6 | 17.7±1.6 | 17.4±1.7 | 0.096 |
| Mean | 18.3±1.9 | 17.6±1.9 | 17.6±1.8 | 18.0±1.8 | 17.9±1.9 | 0.175 |
| *p* | 0.103 | 0.033 | 0.006 | 0.229 | <0.001 |  |
| FML mm |  |  |  |  |  |  |
| NC | 32.3±3.9 | 31.7±3.7 | 31.5±3.0 | 32.2±3.2 | 31.9±3.4 | 0.635 |
| RC | 33.1±4.0 | 33.2±3.7 | 32.8±2.8 | 33.4±2.8 | 33.1±3.3 | 0.845 |
| LC | 31.0±4.0 | 31.1±3.2 | 30.8±2.5 | 31.2±2.9 | 31.0±3.1 | 0.937 |
| Mean | 32.1±4.0 | 32.0±3.6 | 31.8±2.9 | 32.3±3.1 | 32.0±3.4 | 0.840 |
| *p* | 0.080 | 0.019 | 0.002 | 0.009 | <0.001 |  |
| CH mm |  |  |  |  |  |  |
| N | 3.3±0.7 | 3.3±0.7 | 3.2±0.7 | 3.2±0.5 | 3.2±0.7 | 0.838 |
| R | 3.3±0.6 | 3.2±0.8 | 2.8±0.6 | 3.0±0.5 | 3.0±0.6 | 0.036 |
| L | 3.1±0.6 | 3.2±0.7 | 2.9±0.6 | 3.0±0.5 | 3.0±0.6 | 0.168 |
| Mean | 3.2±0.6 | 3.2±0.7 | 3.0±0.6 | 3.1±0.5 | 3.1±0.6 | 0.144 |
| *p* | 0.576 | 0.811 | 0.025 | 0.355 | 0.020 |  |
| ICD mm |  |  |  |  |  |  |
| NC | 22.9±2.4 | 23.0±2.5 | 23.0±2.1 | 23.4±2.2 | 23.0±2.3 | 0.762 |
| RC | 23.7±2.6 | 24.2±2.3 | 23.9±2.2 | 24.6±1.8 | 24.1±2.3 | 0.308 |
| LC | 21.9±2.3 | 22.5±2.1 | 21.9±1.8 | 22.9±1.8 | 22.3±2.0 | 0.110 |
| Mean | 22.8±2.5 | 23.2±2.4 | 22.9±2.2 | 23.6±2.0 | 23.1±2.3 | 0.315 |
| *p* | 0.008 | 0.002 | <0.001 | 0.001 | <0.001 |  |

gH: geometric height; eH: effective height; ComH: commissure height; FML: free margin length; CH: coaptation height; ICD: inter-commissural distance. NC: non-coronary leaflet; RC: right coronary leaflet; LC: left coronary leaflet. NR: between NC and RC; RL: between RC and LC; LN: between LC and NC.

Table S3. Inter and intra age group statistical comparison of ratios for leaflet-root interrelationship

| Leaflet-Root Ratios | Age 1  (n=39) | 2  (n=43) | 3  (n=50) | 4  (n=36) | Mean | *p* |
| --- | --- | --- | --- | --- | --- | --- |
| gH/AD | 0.69±0.06 | 0.69±0.07 | 0.69±0.07 | 0.70±0.07 | 0.69±0.07 | 0.487 |
|  |  |  |  |  |  |  |
| gH/FML | 0.50±0.06 | 0.49±0.05 | 0.48±0.05 | 0.49±0.06 | 0.49±0.06 | 0.365 |
|  |  |  |  |  |  |  |
| eH/gH | 0.57±0.06 | 0.58±0.08 | 0.59±0.07 | 0.58±0.07 | 0.58±0.07 | 0.323 |
|  |  |  |  |  |  |  |
| gH/STJ | 0.59±0.05 | 0.57±0.03 | 0.56±0.04 | 0.56±0.05 | 0.57±0.05 | 0.001 |
|  |  |  |  |  |  |  |
| FML/ICD | 1.41±0.09 | 1.38±0.08 | 1.38±0.08 | 1.37±0.07 | 1.38±0.08 | 0.050 |
|  |  |  |  |  |  |  |
| eH/ComH | 0.49±0.06 | 0.51±0.07 | 0.50±0.06 | 0.50±0.06 | 0.50±0.07 | 0.244 |

gH: geometric height; eH: effective height; ComH: commissure height; FML: free margin length; CH: coaptation height; ICD: inter-commissural distance; STJ: sino-tubular junction (area-derived diameter); AD: annulus diameter.

NC: non-coronary leaflet; RC: right coronary leaflet; LC: left coronary leaflet.

NR: between NC and RC; RL: between RC and LC; LN: between LC and NC.

Table S4. Parameters and ratios stratified by gender

| Variables | Male  (n=87) | Female  (n=81) | *p* |
| --- | --- | --- | --- |
| Age years | 48±15 | 52±15 | 0.074 |
| Annulus area mm^2^ | 435.9±44.3 | 353.3±43.5 | <0.001 |
| STJ area mm^2^ | 647.0±81.8 | 529.9±84.8 | <0.001 |
| STJ/annulus area | 1.50±0.23 | 1.51±0.23 | 0.755 |
| gH mm | 16.2±1.2 | 14.7±1.2 | <0.001 |
| eH mm | 9.2±1.1 | 8.6±1.0 | <0.001 |
| CH mm | 3.3±0.5 | 2.9±0.5 | <0.001 |
| FML mm | 33.8±2.5 | 30.0±2.5 | <0.001 |
| ICD mm | 24.3±1.6 | 21.8±1.6 | <0.001 |
| ComH mm | 18.7±1.6 | 17.0±1.3 | <0.001 |
| gH/AD | 0.69±0.05 | 0.69±0.04 | 0.710 |
| gH/STJ | 0.57±0.05 | 0.57±0.05 | 0.954 |
| eH/gH | 0.57±0.05 | 0.59±0.06 | 0.026 |
| gH/FML | 0.48±0.04 | 0.49±0.04 | 0.165 |
| FML/ICD | 1.4±0.07 | 1.4±0.06 | 0.149 |
| eH/ComH | 0.50±0.06 | 0.51±0.05 | 0.207 |

STJ: sino-tubular junction; AD: annulus diameter; gH: geometric height; eH: effective height; ComH: commissure height; FML: free margin length; CH: coaptation height; ICD: inter-commissural distance.

Table S5. Calculated aortic root and leaflet measures for gH from 12 to 25mm based on linear regression equation.

| gH  (mm) | AD  (mm) | FML  (mm) | ICD  (mm) | STJ  (mm) | ComH  (mm) | eH  (mm) | CH  (mm) |
| --- | --- | --- | --- | --- | --- | --- | --- |
| 12 | 19.5 | 27.3 | 19.7 | 23.8 | 15.5 | 7.2 | 2.48 |
| 13 | 20.3 | 28.6 | 20.7 | 24.8 | 16.2 | 7.7 | 2.66 |
| 14 | 21.2 | 30.0 | 21.7 | 25.9 | 16.8 | 8.2 | 2.84 |
| 15 | 22.0 | 31.3 | 22.6 | 26.9 | 17.5 | 8.7 | 3.01 |
| 16 | 22.8 | 32.6 | 23.6 | 27.9 | 18.2 | 9.2 | 3.19 |
| 17 | 23.6 | 34.0 | 24.6 | 29.0 | 18.9 | 9.7 | 3.37 |
| 18 | 24.4 | 35.3 | 25.6 | 30.0 | 19.6 | 10.2 | 3.55 |
| 19 | 25.3 | 36.7 | 26.6 | 31.1 | 20.3 | 10.7 | 3.73 |
| 20 | 26.1 | 38.0 | 27.6 | 32.1 | 21.0 | 11.2 | 3.9 |
| 21 | 26.9 | 39.3 | 28.6 | 33.1 | 21.7 | 11.7 | 4.08 |
| 22 | 27.7 | 40.7 | 29.5 | 34.2 | 22.3 | 12.2 | 4.26 |
| 23 | 28.5 | 42.0 | 30.5 | 35.2 | 23.0 | 12.7 | 4.44 |
| 24 | 29.4 | 43.4 | 31.5 | 36.3 | 23.7 | 13.2 | 4.62 |
| 25 | 30.2 | 44.7 | 32.5 | 37.3 | 24.4 | 13.7 | 4.79 |

Linear regression equations: AD=9.7+0.819gH; FML=11.2+1.34gH; ICD=7.87+0.985gh; STJ=11.3+1.04gH; ComH=7.23+0.687gH; eH=1.19+0.5gH; CH=0.344+0.178gH
